# Nurse perception of care of hospitalized older adults - a comparative study between northern and central regions of Portugal

**DOI:** 10.1590/1518-8345.0839.2757

**Published:** 2017-10-19

**Authors:** João Paulo de Almeida Tavares, Alcione Leite da Silva, Pedro Sá-Couto, Marie Boltz, Elizabeth Capezuti

**Affiliations:** 1 PhD, RN, Centro Hospitalar e Universitário de Coimbra, Coimbra, Portugal.; 2 Profesor, Department of Health Sciences, University of Aveiro, Aveiro, Portugal.; 3 Profesor Asociado, Department of Mathematics, University of Aveiro, Aveiro, Portugal.; 4 Associate Professor, Penn State College of Nursing, University Park, PA, USA.; 5 Assistant Dean for Research and Director of the Center for Nursing Research, Hunter-Bellevue School of Nursing, Hunter College of City University of New York, New York, NY, USA

**Keywords:** Aged, Geriatric Nursing, Hospitalization, Working Environment

## Abstract

**Objective:**

to analyze the relationship between the perceptions of nurses about geriatric care (GC) environment and geriatric nurses’ knowledge and attitudes according to unit type considering the northern and central regions of Portugal.

**Method:**

a cross-sectional study was developed among 1068 Portuguese’s nurses in five hospitals. The instrument was Geriatric Institutional Assessment Profile - Portuguese version. The independent samples t-test was when the assumption of normality was verified, otherwise, the Mann-Whitney U test was used. The level of significance was 5%.

**Results:**

the profile of perceptions of GC showed a relatively homogeneous pattern (no statistically significant results were found). For the geriatric care environment scale, only the CC/ED units presented significant differences in all considered subscales (resource availability; aging-sensitive care; institutional values; and continuity of care), with more positive perceptions among nurses in the northern region. In Professional Issues scales, only the scale perception of burden related with upsetting behaviors revealed significant differences between regions in all specialties.

**Conclusion:**

the findings suggest the need for increased investment by hospital leaders to promote a geriatric nursing practice environment that supports the specialized needs of hospitalized older adults.

## Introduction 

The Nursing Practice Environment (NPE) is essential for job satisfaction of professional nurses and for their effectiveness in promoting high quality outcomes for inpatients^1^. The NPE in specialized inpatient wards (e.g., oncology, intensive care) has been shown to be an important factor in the perceptions of nurses about their practice and is related to nurse, patient, and organizational outcomes^2^ ^-^ ^4^. Thus, the evaluation of the NPE should consider the specific dimensions of the context to be evaluated^2^ ^-^ ^5^. The delivery of care to hospitalized older adults is shaped by a combination of factors that includes organizational culture, resource availability and work environment, thus reinforcing the importance of measuring the NPE with a specific focus on geriatric care. The research demonstrates that there are significant differences in the evaluation of the NPE when using a general survey versus a survey with geriatric specificity^5^. For this reason, Kim and colleagues^5^ emphasized the need to utilize geriatric-specific indices when evaluating the NPE of nurses caring for hospitalized older adults. Thus, this assessment should focus on structures and processes within the specific dimensions of the geriatric nursing practice environment^2^. 

Considering this specificity, the Nurse Improving Care for Healthsystem Elders program (NICHE) developed an instrument to assess the perception of nurses about care for hospitalized older adults - the *Geriatric Institutional Assessment Profile* (GIAP) or “*Avaliação do Perfil Geriátrico Institucional*” (APGI) in the Portuguese version. The NICHE program is designed to improve geriatric nursing care of hospitalized older adults. An integrative review showed that GIAP has a high degree of specificity, conformity, appropriateness, and utility in the evaluation of nurses’ perceptions of geriatric care, and is a crucial tool in the development and improvement of caring for hospitalized older adults^6^.

An increasing number of studies based on GIAP sought to analyze two central aspects of geriatric nursing care: the Geriatric Nursing Practice Environment (GNPE) and the nurse’s knowledge and attitudes^7^ ^-^ ^11^. The assessment of GNPE examines two dimensions: extrinsic or the Geriatric Care Environment (e.g., institutional values regarding older adults, resource availability, aging-sensitive delivery, and capacity for collaboration) and intrinsic or Professional Issues (e.g., specific points of view to one’s own practice). These factors shape the geriatric institutional milieu^2^. A critical first step in developing system level initiatives to improve the care of the older adult is the evaluation of the geriatric care environment, and organization’s capacity to create systematic change through an assessment of the organizational strengths and readiness to adopt evidence-based geriatric care. 

 In Portugal, the results showed that nurses were dissatisfied with the environment and organizational support for geriatric nursing care^12^ ^-^ ^13^ and demonstrated the low level of knowledge and negative attitudes regarding geriatric syndromes (pressure ulcers, incontinence, restraint use, and sleep disturbance)^14^. 

Portugal, as other European countries, is organized by regions (Nomenclature of Units for Territorial Statistics) for referencing the subdivisions of countries. This categorization of subdivisions is used for statistical purposes and corresponds to administrative division within each country. In Portugal, the healthcare system is organized into five regional health administrations (RHAs), two of these are the northern and the central RHA. According to the data from the National Statistical Institute^15^, the central region has a higher aging index (163.4%) and a lower number of nurses working in the hospitals (8,064) than the northern region (aging index - 113.3% and 11 813 nurses in the hospitals). These differences could influence the nurses’ perception of institutional milieu and nurses’ geriatric practice and nurses’ perception about geriatric care. Additionally, the quality geriatric care requires a NPE in which the structure and processes of hospital services focus on specific patient care needs and the importance of the institutional-level support the unit-level planning, implementation, and evaluation of initiatives to improve institutional milieu. Considering this, the purpose of this study was: 1) analyze the relationship between the perceptions of nurses about GNPE (intrinsic and extrinsic factors) according to unit type considering the northern and central regions of Portugal; 2) analyze the relationship between geriatric nurses’ knowledge and attitudes according to unit type considering again the northern and central regions of Portugal.

## Methods 

This study utilized a quantitative, exploratory-descriptive, cross-sectional and correlational approach.

### Data and sample

This study was conducted in five hospitals; two located in the northern region (Oporto) and three in the center of the country (Coimbra and Aveiro). Of these hospitals, three are hospital centers and two are academic hospitals. In order to ensure a diverse sample, the selection of these hospitals was based on bed size as well as the number of inpatients and nurses^14^. The geographical proximity of these hospitals was also considered in order to optimize data collection^14^. 

The method of sampling was nonprobability convenience and included all registered nurses who worked in medical or surgical specialty units, critical care units (CCUs) and emergency departments (ED). We excluded nurses who worked on units serving primarily younger adults or children, and nurses who did not provide direct care (including nurse managers and supervisors). The project was submitted to and approved by the ethics committees of each of the 5 hospitals; all of the participants signed an informed consent form.

### Data collection

The researcher, in partnership with the nurse manager of the units, conducted the data collection. The steps taken in this process are described in the article of validity of the GIAP^14^. Among the 2271 surveys distributed, 1173 were returned, representing a 52% response rate, which is considered acceptable^16^. However, 105 were not completed and were excluded. Thus, the final sample included 1,068 registered nurses. 

### The survey

The GIAP is a 152-item self-completed survey that examines unit/hospital and respondent demographic/professional characteristics and includes eight major scales: Geriatric Nursing Knowledge/ Attitudes Scale - 23 items (nurse´s knowledge and attitudes toward four common geriatric syndromes: pressure ulcers, incontinence, restraint use, and sleep disturbance). The Geriatric Care Environment (GCE) Scale - 27 items (organizational characteristics that promote or hinder the GCE) - composite of the following 4 subscales: resource availability (perceptions of: access to human and material resources specific to care of older adults and management support of communication with patients and families), aging-sensitive care delivery (geriatric specific, evidence-based, individualized care that promotes informed decision making, and is continuous across settings), institutional values regarding older adults (perceptions of: respect for the rights of older adults, involvement of older adults and families in decision making, and support of nurse autonomy and personal growth) and staff and continuity of care (perceptions of : continuity of care between different wards and organization). The Professional Issues (PI) scales - 42 items (interpersonal and coordinative aspects of professional practice), composite of the following 6 scales: staff/family/patient disagreement regarding treatment of common geriatric syndromes; perceived legal vulnerability related to pressure ulcers, falls, restraint use, nosocomial infection and injuries related to sedating medication; burden of upsetting behaviours; staff disagreement around treatment of common geriatric syndromes; perceived upsetting behaviours, and use of geriatric services (perception of the appropriate use of geriatric specialists and professional practices). The survey uses Likert scales (ranging from 0 to 4 points). Higher scores indicate a favorable GNPE and better geriatric nurse knowledge and attitudes. An integrative review of this instrument describes the strengths and weaknesses of GIAP and their implications for geriatric nursing practice^6^.The translation, adaptation, and validation of the GIAP scales to Portuguese^12^ were based on the standards of the *International Society for Pharmacoeconomics and Outcomes Research* (ISPOR) *Task Force for Translation and Cultural Adaptation* ^17^. The internal consistency reliability (Cronbach’s alpha) ranges from 0.601 to 0.919, and thus shows good to very good internal consistency, similar to other studies^7^ ^-^ ^8^.

### Data analysis

Descriptive and inferential statistics were used to systematize and enhance the information provided by the data, including: frequencies, measures of central tendency and variability. The independent samples t-test was used to compare the gathered results from the northern and central regions in CCU/ED, medical and surgical units. The normality (determined by visually inspecting Q-Q plots) and homogeneity (Levene’s test) conditions were assessed. When the assumption of normality was not verified, the Mann-Whitney U test was used. All of the statistical analyses were performed using Predictive Analytics Software (PASW) Statistics 18 (IBM Corporation, Armonk, NY) with a level of significance set at 5%.

## Results

### Sites and samples

The sample (n=1068) was mostly comprised of female nurses (79.7%) who were single (45.4%) or divorced (34.3%). The mean age of the nurses was 34.1 years (SD=8.5). The nurses in the sample reported an average of 11.3 years (SD=8.4) of work experience, 10 years (SD=8.1) working in their institution and 7.5 years (SD=6.5) working in their units. 

The overall majority of the nurses had a college degree in nursing (N=949; 88.8%) followed by the nurse specialist’s degree certificate (Portuguese’s Nursing Council) (N=119; 11.2%). In terms of postgraduate academic education, 14.1% had specialization and 6.8% had a master’s or doctoral degree. The majority of the nurses (N=922; 86.3%) reported not having received any gerontological education or training; 94 (8.8%) had participated in short courses (continuing education), and 52 (4.9%) had received training in an academic program (master’s or doctoral degree)^14^.

### Geriatric nursing knowledge and attitudes 

The results indicated in Table 1 demonstrate no significant differences (p>0,05) for both total score and subscales on Geriatric Knowledge and Attitudes between regions in the three units considered. 

**Table 1:** Geriatric Knowledge/Attitude, Geriatric Care Environment & Professional Issues by Unit: Northern and Central, Portugal, 2011 (M*=SD^†^)

	**CCU/ED** ^**‡**^ **(N=201)**		**Medical units** **(N=536)**		**Surgical units** **(N=331)**	
	**North** **(N=15)**	**Central** **(N=186)**	**North** **(N=231)**	**Central (N=305)**	**North** **(N=129)**	**Central (N=202)**
**Geriatric Nursing Knowledge/Attitudes**						
**Knowledge**	**0.37=0.11**	**0.4=0.14**	**0.41=0.14**	**0.43=0.13**	**0.42=0.14**	**0.41=0.14**
**Attitudes**	**0.38=0.23**	**0.28=0.19**	**0.41=0.2**	**0.39=0.19**	**0.42=0.2**	**0.38=0.17**
**Total score**	**0.38=0.11**	**0.36=0.12**	**0.41=0.12**	**0.41=0.12**	**0.43=0.13**	**0.41=0.12**
**Geriatric Care Environment**						
**Resource Availability**	**21.5=9.0** ^**§**^	**14.8=8.8** ^**§**^	**17.0=8.0**	**17.7=8.3**	**17.8=8.4**	**18.0=7.9**
**Aging-sensitive Care Delivery**	**16.1=6.4** ^**§**^	**11.6=6.8** ^**§**^	**16.6=5.2**	**15.8=5.3**	**16.4=5.9**	**16.3=5.0**
**Institutional Values**	**11.7=3.5** ^**§**^	**9.1=4.9** ^**§**^	**12.8=4.8** ^**§**^	**11.5=4.7** ^**§**^	**13.4=5.3** ^**§**^	**11.3=4.4** ^**§**^
**Continuity of care**	**5.6=2.8§**	**3.7=2.7§**	**5.7=2.5**	**5.2=2.6**	**5.4=2.6**	**5.6=2.3**
**Score total**	**54.4=20.3** ^**§**^	**39.0=18.7** ^**§**^	**52=15.5**	**50.2=15.7**	**52.9=16.3**	**51.1=14.5**
**Professional Issues**						
**Staff/Family/Patient Disagreement**	**3.4=0.6**	**3.1=0.7**	**3.1=0.7** ^**§**^	**3.2=0.7** ^**§**^	**3.3=0.7**	**3.2=0.6**
**Perceived Legal Vulnerability**	**2.6=1.1**	**2.2=1.0**	**2.5=0.9**	**2.4=1.0**	**2.5=0.9**	**2.3=0.9**
**Burden of Upsetting behaviors**	**0.7=0.5** ^**§**^	**1.0=0.5** ^**§**^	**0.7=0.6** ^**§**^	**1.1=0.4** ^**§**^	**0.8=0.6** ^**§**^	**1.1=0.5** ^**§**^
**Staff disagreement**	**3.5=0.6** ^**§**^	**3.1=0.7** ^**§**^	**3.2=0.6**	**3.1=0.7**	**3.2=0.7**	**3.2=0.7**
**Perceived Upsetting behaviors**	**0.8=0.4**	**0.8=0.4**	**0.7=0.3**	**0.7=0.3**	**0.8=0.4**	**0.8=0.3**
**Use of Geriatric Services**	**0.1=0.3**	**0.3=0.4**	**0.3=0.6** ^**||**^	**0.5=0.6** ^**||**^	**0.4=0.7**	**0.4=0.5**
**Total score**	**95.3=18.8**	**89.4=16.8**	**89.5=15.5** ^**§**^	**92.2=15** ^**§**^	**92.5=17.0**	**93.7=14.6**

*Mean

†Standard Deviation

‡Critical Care Units/Emergency Department

§Calculated values using *Independent Samples t-Test* (p<0.05)

||Calculated values using *Mann*-*Whitney U*-*test* (p<0.05)

### Geriatric Care Environment 

The results concerning the GCE - extrinsic factors (Table 1) showed statistically significant differences on both total scores and all subscales between regions only among nurses working in the CCU/ED. Additionally, statistically significant differences were found on some subscales in medical and surgical units. The nurses who worked in these units in the northern region had significantly higher mean values (more positive perception) compared to nurses who worked in the central region (t(199)=2.96, p<0.01). For the other units, the overall score showed no statistical differences (p>0.05). In the CCU/ED unit, significant differences were found in all subscales, with more positive perceptions among nurses in the northern region (resource availability: t(199)=2.85, p<0.01; aging-sensitive care: t(199)=2.46, p=0.02; institutional values: t(199)=2.12, p<0.04; continuity of care: t(199)=2.56, p=0.01). 

In the other units (GCE - extrinsic factors, Table 1), only the subscale *institutional values regarding older adults* showed significant differences between the two regions. Nurses who worked in the northern region in medical wards (t(534)=3.72, p<0.01) and surgical wards (t(328)=3.84, p<0.01) had a more positive perception of: respect for the rights of older adults, involvement of older adults and families in decision making, and support of nurse autonomy and personal growth. 

### Professional issues 

The results on the overall score of GCE - intrinsic factors (Professional Issues scales, Table 1) showed only statistically significant differences between regions in the medical unit (t(516)=-2.01, p=0.04). Nurses who worked in the central region reported fewer barriers to optimal care. In other units, the overall score showed no significant differences (p>0,05).

The nurses *perception of burden related with upsetting behaviors* scale revealed significant differences between regions in all specialties (CCU/ED: t(199)= 3.05, p<0.01; Medical: t(534)=-9.06, p<0.01; Surgical: t(329)= -4.89, p<0.01) (Professional Issues scales, Table 1). Nurses who worked in the center of the country were less likely to perceive upsetting behaviors among older patients, and these behaviors did not represent significant barriers to providing ‘good’ quality of care. 

Regarding medical units (Professional Issues scales, Table 1), the scales *staff/patient/family disagreements* (t(534)=-2.61, p<0.01) and *use of geriatric services* (t(534)=3.72, p<0.01) showed significant differences between the nurses who worked in northern and central regions. The nurses from the central region had significantly higher mean scores for *staff/patient/family disagreements* (experienced fewer disagreement among staff/family/staff). On the scale *use of geriatric services*, nurses from the northern region reported more barriers in the incorporation of geriatric resources in their practice. 

Finally, statistically significant differences were found on the *staff disagreement* scale between nurses who worked in the northern and central regions in the CCU/ED unit (t(199)=2.09, p=0.04) (professional issues scales, Table 1). Nurses who worked in the northern region reported fewer disagreements among staff compared to those from the central region.

## Discussion

Studies of GNPE have emerged in the literature over the last decade, driven by research conducted in North America. In Portugal, studies demonstrate that the perception of the geriatric milieu among nurses is considered unsatisfactory^11^ ^-^ ^12^. Analyzing the differences between the northern and central regions, this study found that nurses who work in the CCU/ED units in the northern region have more positive perceptions of the geriatric care environment, the extrinsic factors of the GNPE. Thus, these nurses had a more positive perception about the organizational commitment to quality geriatric care. A younger population and a higher patient to nurse ratio in this region could influence their perceptions of the geriatric care environment, especially in these units^12^. Aspects such as the emphasis on teamwork that promotes more effective communication between professionals^18^ may contribute to these results. In addition, the medical teams in the emergency services in the northern region do not rotate every day, which may enable greater collaboration, knowledge sharing, and autonomy of nurses. However, the number of nurses in the CCU/ED in the northern region is very small and likely could influence these results. 

There were no significant differences between medical and surgical units in the total score and in the three subscales of GNPE (*resource availability*, *aging-sensitive care,* and *continuity of care*). Consequently, there is a relatively homogeneous pattern in these units, which could suggest that the difference in the number of hospitalized older adults and nurse to patient ratios apparently do not influence nurses’ perceptions about the geriatric care environment. 

These results show the importance of developing professional standards to guide the GNPE, as well as the clear commitment of nursing leaders to incorporating the best practices in caring for hospitalized older adults who have unique and complex needs^7^. The results in medical and surgical units further suggest the importance of decision-makers of RHAs and hospital leaders in effectively implementing management measures such as: a) promoting collaboration among the disciplines (interdisciplinary, using geriatric protocols, management of conflicts that emerged in caring for older adults); b) promoting geriatric education and training among healthcare professionals and equipment and resources tailored to older adults; c) focus on patient/family-centered care for older adults; d) promoting evidenced-based geriatric nursing protocols for best practice; e) definition of institutional policies based on the care needs of hospitalized older adults; f) establishment of partnerships with other healthcare and/or social institutions to promote effective, efficient, and safe continuity of care. These results are supported by another study^7^ which emphasized the need for comprehensive organizational support for the GNPE. 

However, it is noteworthy that in the northern region, in all units, nurses have more positive perceptions regarding the subscale *institutional values*. Considering the content of this subscale, these results could suggest that, when compared to the central region organizations, the northern institutions may demonstrate a greater commitment to promoting the autonomy of nurses, as well as their professional development. This commitment to becoming *learning organizations* is evident in their support for nurses’ professional development, lifelong learning, and mutual sharing of knowledge^19^.

The perception of barriers to the development of quality care for older adults is not associated with geographical location of the CCU/ED and surgery units. Furthermore, in medical specialties, there are significant differences between the two regions. The high number of hospitalized older adults in these wards could contribute to nurses’ awareness and experience of more barriers that may compromise the quality of care of these patients. 

The nurses who worked in medical units in the northern region perceived more barriers to providing a good quality of care to hospitalized older adults. Thus, it becomes important to implement programs that may overcome some of these barriers, especially those associated with the perception of upsetting behaviors, ineffective communication between staff, older adults and family, and a shortage of geriatric resources. 

This study also suggests that, regardless of units, those in the northern region report that burden of upsetting behaviors and lack of geriatric resources are potential barriers to promoting quality patient care and influence hospital care of older adults. These aspects seem to demonstrate that nurses who worked in this region could be more aware of these potential barriers and their influence upon care delivery.

Nurses working in CCU/ED units in the northern region reported less *staff disagreement* (fewer barriers to optimal care). One possible explanation is that the medical staff do not rotate frequently, thus promoting better communication and collegial relationships between nurses and physicians. Positive working relationships are considered essential to ensure safety and quality care in this type of unit^19^.

The Professional Issues scales, *perception of upsetting behaviors* and *legal vulnerability,* showed no significant differences between regions. On the other hand, the *perception burden of upsetting behaviors*, showed statistically significant differences between regions in all specialties. These results suggest that a barrier to quality care may be due not to the prevalence of these behaviors but to how nurses deal with this situation. These findings corroborate the findings from other studies that analyzed the development of nursing care to persons with dementia, including patients who demonstrated behavioral manifestations of distress^20^ ^-^ ^22^. Thus, the education of nurses in caring for hospitalized older adults with dementia should be considered a priority, because reducing the burden of these professionals could contribute to improving the outcomes for patients and nurses. 

Based upon the *legal vulnerability* scale results, the results may suggest that nurses in this study are not acquainted with the legal issues related to pressure ulcers, falls, physical restraint and nosocomial infection. In contrast, a study performed in North America found that the responses to this scale were the most negative, constituting one of the main barriers to providing quality of care among the nurses^11^. Sociocultural differences, professional regulation, and the legal system, could explain the different perceptions of nurses in these two countries.

The findings showed no significant regional differences in the *geriatric knowledge and attitudes scales*. These results were not completely unexpected given the lack of geriatric content in nursing curricula in Portugal as well as the few continuing education offerings or available specialization in geriatric care. Additionally, there are no geriatric nursing membership organizations such as NICHE in Portuguese hospitals that promote geriatric staff education. The lack of nurse’s knowledge and negative attitudes can influence the nurse´s awareness about the important of the organizational leadership developed a comprehensive geriatric programs tailored to the specificity’s of the medical, surgical and departments CCU/ED units.

The knowledge and attitudes of nurses may have other dimensions that go beyond the region and units. The low level of knowledge and negative attitudes of nurses of hospitalized older adults^14^ may be related to the training of nurses in Portugal since this has not followed the demographic and epidemiological changes of the last decades. These results could reflect a lack of investment in geriatric nursing care by the nursing schools, professional nursing organizations, hospital administrations, and governmental policies. In both regions, the training of nurses should reflect the current and future needs of care to respond to the aging Portuguese population who are most likely to receive acute care services^22^ ^-^ ^23^.

### Limitations

The study has limitations. There is an imbalance in sample size across all types of units, especially in the CCU/ED unit where this imbalance is most evident, being higher in the central region, although the statistical tests (parametric and nonparametric) reported similar results. Future studies should seek to include more homogeneous samples with respect to size. The convenience sample, location of hospitals (north and center of the country), and the exclusion of hospitals with less than 300 beds, limit the generalizability of the results to other regions of the country and to other types of hospital with smaller dimensions. The inclusion of different hospitals and extending the research to other regions of the country would have increased the diversity of the sample and generalizability of the results.

Another limitation is that all data were collected by self-report and could be subject to respondent bias (e.g., nurses who are more dissatisfied may be more likely to respond negatively to the GNPE scales). Furthermore, these results could be influenced by the specific and unique characteristics of units and organizational culture and climate, which could affect the perception of these professionals. Thus, a more detailed categorization of the GNPE could provide additional information and a better understanding of the perception of nurses regarding the environment in which they develop their geriatric practice. 

## Conclusion 

The aging population in Portugal continues to impose a major impact on healthcare services and the development of care for older adults. The evaluation of the geriatric care profile is crucial to ensure nursing quality of care. Although there are some statistical differences between GNPE and geriatric nurses’ knowledge and attitudes according the northern and central regions of the country, considering the units analyzed, for the most of the dimensions of the GIAP scales, there were no relationships, pointing to a relatively homogeneous profile about the geriatric nursing care. This study highlights: 1) the importance of greater commitment and support of hospital leaders in both regions of the country; 2) the need for systematic changes in the GNPE in the north and center of the country; and 3) increased investment in continuous training and expertise of nurses in geriatric care. This study opens a new field of study about the nursing care of hospitalized older adults, with important implications for practice, management, education, and research. It is expected that these results will contribute to the developing, planning, and implementation of geriatric care models or programs to promote the quality of care in these two regions of Portugal. 
